# What are the kinematic characteristics of the world champion couple in competitive ballroom dance during the waltz's spin movement?

**DOI:** 10.3389/fspor.2022.941042

**Published:** 2022-07-22

**Authors:** Yasuyuki Yoshida, Arunas Bizokas, Katusha Demidova, Shinichi Nakai, Rie Nakai, Takuichi Nishimura

**Affiliations:** ^1^Human Augmentation Research Center, National Institute of Advanced Industrial Science and Technology, Chiba, Japan; ^2^Non-affiliated, Fairfield, CT, United States; ^3^Dance Jardin, Tokyo, Japan; ^4^School of Knowledge Science, Japan Advanced Institute of Science and Technology, Ishikawa, Japan

**Keywords:** kinematics, spin, waltz, biomechanics, ballroom dance

## Abstract

To evaluate competitive ballroom dancing, one of the official events of the World Games, spin movements used by advanced dancers who ranked high in actual competition may be employed. The purpose of this study was to investigate the kinematic characteristics of spin movements in competitive ballroom dancing performed by world champion couples, especially holding posture and lower limb movements. A champion couple and 13 national-level competitive ballroom dancers as the control group participated in this study. An inertial measurement unit (IMU) system (MVN Link; Xsens, Netherlands) consisting of 17 IMUs, attached to feet, shanks, thighs, pelvis, sternum, head, upper arms, forearms, and hands of the dancers, was used to obtain three-dimensional kinematic data at 240 Hz. The overall trend was that the actual and normalized stride lengths of the champion male and female dancers tended to be longer than those of the male and female dancers in the control group. Further, large differences were observed in the pelvic and rib cage segments movements, and the relative angle of the rib cage segment to the pelvic segment. The champion male dancer started to move from the pelvic segment, whereas the champion female dancer and the national-level top male and female dancers started to move from the rib cage segment. During the spin movement, the champion male dancer was in a position where the rib cage segment was rotated to the left with respect to the pelvic segment, whereas the other dancers were in a position where the rib cage segment was rotated to the right. Although limited to technical aspects, these dance kinematic characteristics of the world champion couples and their differences from those of the control group dancers, will be helpful to competitive ballroom dancers and coaches in their daily practice.

## Introduction

Competitive ballroom dancing (DanceSport) is an official event in the world games, and is characterized by formation of pairs of males and females in closed-hold position (Vaczi et al., 2016). In a pair, male and female partners face each other, with overlapping of only the right half of the body; the pelvis, thighs, arms, and hands of male and female partners remain in contact. Despite a complicated posture, advanced dance couples are able to move smoothly and rapidly. Further, our understanding of the biomechanics of competitive ballroom dancing is limited (McCabe et al., 2013). Therefore, it is novel to study the biomechanics of the closed-hold position and its associated movements during dance steps in competitive ballroom dance.

Evaluating a competitive ballroom dance performance is difficult in comparison to that of other competitive sports where performance is determined on the basis of measurements of physical quantities, such as time, distance, and mass. The World DanceSport Federation has listed technical quality, movement to music, partnering skills, and choreography and presentation as the main components for the evaluation of competitive ballroom dance performances (Premelc et al., 2019); however, none of these components can be measured using physical quantities (WDSF, 2015). Hence, to better understand the biomechanics associated with this competitive dance form, we extracted various dance movements characteristics of dancers who are ranked high in the actual competitions. We reveal identification of a variable that can be implemented to evaluate competitive ballroom dance performances. This approach of employing champions (top-ranked experimental participant) in a specific field for conducting biomechanics analyses (Fujii and Moritani, 2012; Whiteside et al., 2013; Hobara et al., 2019) is used previously. We also included the world champion couples in competitive ballroom dancing (Yoshida et al., 2020, 2021) in our previous biomechanics studies.

In a previous study, Prosen et al. (2013) mounted a camera on the ceiling of a competitive ballroom dance venue and analyzed the movement trajectory, total time, and average speed during the Viennese waltz dance style. The previous study showed that the average speed of natural turns (indicating clockwise rotation) was significantly different between the top and the other ranks of dancers, although auditory elements and musical connotation were not considered. However, detailed information on the lower limb movements related to speed during dancing is lacking and, therefore, further kinematic studies are needed.

According to the World DanceSport Federation, spin is a type of turn (WDSF, 2013). Advanced dancers frequently use spin movements during the competitive ballroom dancing. In our previous kinematic study (Yoshida et al., 2020, 2021), we analyzed the basic movements of waltz dancers as they turned ~135° while moving three steps in three beats. A typical waltz spin movement was ~315° in the first three beats and two revolutions in the next three beats. Furthermore, spin movement was continuously performed after that. Higher level competitive ballroom dancers can perform movements that require such a large amount of rotation in a short time. Kinematic studies of competitive ballroom dancers during such dance movements have not been conducted.

The purpose of this study was to investigate the kinematic characteristics of spin movements in competitive ballroom dancing performed by world champion couples, especially holding posture and lower limb movements. We hypothesized that the champion couples would show remarkable differences in some of the kinematic variables than that of the control group.

## Methods

### Participants

A couple that held the world championship in ballroom dancing for 10 years was included in this study. They were still a world champion couple at the time of measurement. The age (years), height (cm), and body mass (kg) of the male/female champion dancers were 40/40, 183/169, and 76/52, respectively. Further, 13 top-ranked national-level competitive ballroom dance couples participated as the control group. The age, height, and body mass (mean ± standard deviation) of the control group were 24.3 ± 5.6 years, 173.4 ± 6.3 cm, and 61.1 ± 4.9 kg for male dancers, and 22.6 ± 4.7 years, 161.2 ± 6.1 cm, and 47.6 ± 5.0 kg for female dancers, respectively. Dance experience as a couple for the control group was 4.4 ± 4.1 years. All couples were certified as grade A or B by the national competitive ballroom dance organization at the time of measurement. In the standard division of The Imperial Highness Prince Mikasa Cup, which is considered to be the most prestigious amateur competition in Japan, seven couples were finalists or semi-finalists. All participants were free of injury and wore comfortable dance shoes. These participants were the same who participated in our previous study (Yoshida et al., 2020, 2021).

### Instruments

An inertial measurement unit (IMU) system (MVN Link; Xsens, Netherlands) (Jurkojc et al., 2017) consisting of 17 IMUs was used to obtain three-dimensional kinematic data at 240 Hz for both the male and female dancers. The IMUs were attached to feet, shanks, thighs, pelvis, sternum, head, upper arms, forearms, and hands of the dancers. This system was time-synchronized and could simultaneously measure the movements of both the male and female dancers at the same time. The IMUs were attached to specialized Lycra suits.

### Experimental setup

The IMUs were attached to the dancers' feet, shanks, thighs, pelvis, sternum, head, upper arms, forearms, and hands. The English waltz song “Without You” was played from the album “Ballroom Symphony” (Casa Musica, Germany); tempo was 28 bars per minute. To build the Xsens rigid 23-link model, anthropometric data were measured for each participant. Further, shoes were worn, the upright posture (N-pose) and walking movements were recorded for model calibration.

### Protocol

Dance couples danced a trial with music as follows (numbers in parentheses are beats): (1) second half of natural spin turn (123), (2) continuous spin (1 and 23), and (3) turning rock to right (1 and 23). The trial was repeated five-times with closed-hold position. There was an interval of ~2 min between the trials.

### Data analysis

**Figure 1:**
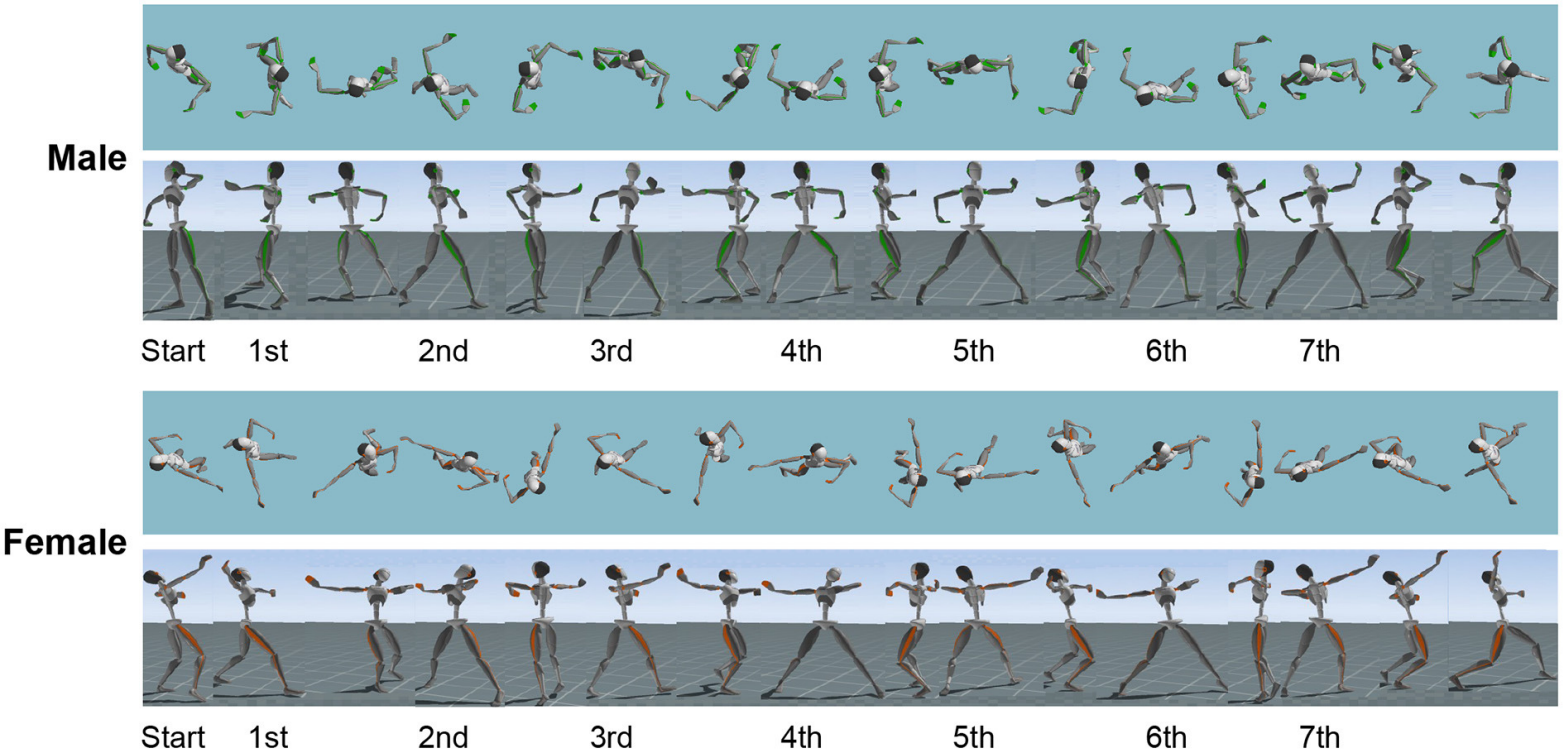
Explanation of spin movement in waltz. The steps from the start to the 7th step are shown. Top: Male, Bottom: Female.

For each trial, data from the second half of the natural spin turn to the end of the continuous spin were used for the analysis. Figure 1 shows the division of the phases. Figure 1 also shows the “start,” which is the start of the analysis section, and the “7th,” which is the end. Each phase was distinguished as a key event by the maximum and minimum step lengths, which was defined as the distance between the right and left ankle positions on a horizontal plane. The count of the second half of the natural spin turn was 123, and consisted of three steps. The counts of the continuous spin were 1 and 23, and consisted of four steps. In total, the spin movement was divided into seven phases.

In the beginning, the male dancers stood with their right leg as a support leg in a backward position with a minimum step length. The first step began with the left toe sliding backward until the maximum left-leg step length was achieved. The second step began from the end of the first step to the maximum right leg step length with right heel contact. The third step began from the end of the second step to the maximum left leg step length with left toe contact and heel raised. Approximately 315° of turning was performed during these three steps of three counts.

This was followed by the movement of the left foot of the third step from backward to forward, leading to the fourth step. The fourth step began from the end of the third step to the maximum right leg step length with right heel contact. The fifth step began from the end of the fourth step to a maximum left leg step length with left toe contact and heel raised. The counts were “1” and “&.” “&” meant that one step was half a beat. Thus, ~1 revolution occurred during the two steps of each count.

The left foot of the fifth step subsequently went from backward to forward and led to the sixth step. The sixth step began from the end of the fifth step to the maximum right leg step length with right heel contact. The seventh step began from the end of the sixth step to the maximum left leg step length with left toe contact and heel raised. Thus, ~1 revolution was made during the two steps of these two counts. Therefore, during spin movement, ~2 revolutions and 7/8 revolutions, accompanied by the distance traveled, were performed.

As male and female dancers moved to face each other during the spin movement, the right-and left-side movements of the male corresponded to the left-and right-side movements of the female partner, respectively. Further, forward and backward movements of the male dancers corresponded to the backward and forward movements of the female dancers, respectively.

Software attached to the motion capture system (MVN Analyze 2019; Xsens, Netherlands) was used for analysis. The step length for the phase division was normalized by the dancer's leg length. The durations required for each phase were also calculated. The Euler angles of each segment and each joint were calculated in three dimensions in the rigid link model. Based on the three components of the velocity vector for each segment, the resultant velocity was calculated using the Pythagorean theorem. The heights of the left and right heels at each step timing were calculated. The average of the five trials of the variable was determined for each participant. A one-sample *t*-test was used to assess the differences in the kinematic variables of each phase between the champion male or female dancer and male or female dancers. Statistical analysis was conducted using the R software (ver. 4.1.1; R Foundation, Vienna, Austria). Statistical significance was set at *p* < 0.05.

## Results

Table 1 shows the actual and normalized step lengths of the dancers during the spin movements that were divided into seven steps. The actual step lengths of the champion male and female dancers were significantly longer than that of the control group male and female dancers, respectively, except for the sixth step in male and fourth step in female. Furthermore, the normalized step length of the champion male and female dancers were significantly longer than that of the control group male and female dancers, respectively, except for the second and fourth steps.

**Table 1:** Absolute and normalized start to seventh step lengths of the champion male and female dancers and the control group male and female dancers during the spin movement.

		**Male dancers**		**Champion male dancer**			**Female dancers**		**Champion female dancer**		
**Step**	**Unit**	**Mean**	**SD**	**Mean**	**SD**	***p*** **-value**	**Mean**	**SD**	**Mean**	**SD**	***p*** **-value**
Start	m	0.38	0.08	0.47	0.03	0.001	0.40	0.09	0.52	0.06	<0.001
1st	m	0.83	0.12	1.04	0.03	<0.001	0.71	0.10	0.87	0.01	<0.001
2nd	m	0.70	0.08	0.78	0.05	0.003	0.89	0.09	0.96	0.01	0.025
3rd	m	0.85	0.07	1.00	0.02	<0.001	0.63	0.08	0.74	0.02	<0.001
4th	m	0.71	0.07	0.77	0.05	0.006	0.95	0.09	0.99	0.02	0.157
5th	m	0.91	0.09	1.11	0.01	<0.001	0.66	0.09	0.83	0.02	<0.001
6th	m	0.66	0.05	0.65	0.03	0.304	0.93	0.10	0.98	0.02	0.092
7th	m	0.82	0.05	0.97	0.01	<0.001	0.57	0.05	0.62	0.02	0.005
Start	% Leg Length	43.14	7.96	49.27	2.63	0.017	45.28	9.65	57.69	6.69	0.001
1st	% Leg Length	94.68	10.20	109.21	3.25	<0.001	79.79	11.02	95.20	1.47	<0.001
2nd	% Leg Length	80.04	8.12	82.43	5.23	0.309	100.94	9.35	105.21	1.21	0.126
3rd	% Leg Length	97.45	4.60	104.94	2.54	<0.001	71.45	8.32	81.19	1.74	<0.001
4th	% Leg Length	81.30	8.30	81.53	4.96	0.925	107.41	9.51	108.86	2.15	0.593
5th	% Leg Length	103.28	7.32	116.56	1.04	<0.001	74.49	8.33	91.49	1.72	<0.001
6th	% Leg Length	75.85	6.17	68.16	2.99	0.001	104.92	10.83	107.72	1.88	<0.001
7th	% Leg Length	94.24	5.81	102.25	1.16	<0.001	64.67	4.43	68.32	2.01	0.012

Table 2 shows the height of the dancer's left and right heels during the spin movement at the timing of the seven steps. In the right heel, the height of the champion male dancer was significantly higher than that of the male dancers in the control group at the first step (*p* = 0.021), the third step (*p* = 0.001), the fourth step (*p* = 0.041), and the seventh step (*p* = 0.026). In the left heel, the height of the start (*p* = 0.001), first (*p* < 0.001) and second (*p* < 0.001) steps for the champion male dancer were significantly higher than those of the control group male dancers. In the right heel, the height of the champion female dancer was significantly higher than that of the female dancers in the control group at the start (*p* = 0.050) and the second (*p* < 0.001) step. In the fourth step, the height of the control female dancer was significantly higher than that of the champion female dancer (*p* = 0.038). In the left heel, the height of the start (*p* = 0.003), first (*p* = 0.001) and sixth (*p* = 0.001) steps of the champion female dancer were significantly higher than those of the female dancer in the control group.

**Table 2:** Left and right heel heights at the timing of each step of the champion male and female dancers and the control group male and female dancers during the spin movement.

			**Male**		**Champion**			**Female**		**Champion**		
			**Dancers**		**Male dancer**			**Dancers**		**Female dancer**		
**Heel**	**Step**	**Unit**	**Mean**	**SD**	**Mean**	**SD**	***p*** **-value**	**Mean**	**SD**	**Mean**	**SD**	***p*** **-value**
	Start	m	0.003	0.008	0.007	0.002	0.125	0.101	0.021	0.114	0.009	0.050
	1st	m	0.004	0.007	0.009	0.004	0.021	−0.002	0.011	−0.004	0.003	0.529
	2nd	m	0.002	0.006	0.004	0.001	0.398	0.044	0.022	0.085	0.013	<0.001
Right	3rd	m	0.095	0.047	0.148	0.012	0.001	0.008	0.011	0.009	0.005	0.651
	4th	m	0.003	0.005	0.006	0.006	0.041	0.089	0.034	0.067	0.007	0.038
	5th	m	0.102	0.048	0.128	0.013	0.068	0.010	0.016	0.012	0.003	0.636
	6th	m	0.006	0.017	0.003	0.001	0.569	0.095	0.039	0.104	0.009	0.449
	7th	m	0.121	0.051	0.157	0.007	0.026	0.040	0.021	0.037	0.006	0.619
	Start	m	0.143	0.027	0.177	0.007	0.001	0.008	0.004	0.012	0.001	0.003
	1st	m	0.049	0.021	0.094	0.010	<0.001	0.025	0.015	0.043	0.009	0.001
	2nd	m	0.102	0.042	0.166	0.010	<0.001	0.076	0.018	0.077	0.007	0.847
Left	3rd	m	0.068	0.023	0.070	0.011	0.839	0.090	0.037	0.084	0.011	0.581
	4th	m	0.122	0.047	0.114	0.018	0.585	0.069	0.021	0.079	0.011	0.130
	5th	m	0.069	0.018	0.059	0.012	0.081	0.126	0.037	0.116	0.012	0.372
	6th	m	0.114	0.027	0.128	0.024	0.082	0.068	0.017	0.088	0.005	0.001
	7th	m	0.102	0.032	0.094	0.008	0.384	0.073	0.020	0.080	0.017	0.210

Table 3 shows the durations required for each step; the first step was significantly longer for the control group male (*p* = 0.006) and female (*p* = 0.002) dancers than that of the champion male and female dancers, respectively, while the second and third steps were significantly longer for the champion male (*p* = 0.015 and *p* = 0.042) and female (*p* = 0.004, *p* < 0.001) dancers than that of the control group male and female dancers, respectively. Further, the duration of the fifth step was significantly longer in control group female dancers than that of the champion female dancer (*p* < 0.001).

**Table 3:** Duration of each step for the champion male and female dancers and the control group male and female dancers during the spin movement.

		**Male dancers**		**Champion male dancer**			**Female dancers**		**Champion female dancer**		
**Step**	**Unit**	**Mean**	**SD**	**Mean**	**SD**	***p*** **-value**	**Mean**	**SD**	**Mean**	**SD**	***p*** **-value**
1st	s	1.25	0.22	1.04	0.08	0.006	0.62	0.22	0.38	0.03	0.002
2nd	s	0.67	0.09	0.74	0.04	0.015	0.63	0.05	0.68	0.03	0.004
3rd	s	0.66	0.08	0.71	0.03	0.042	0.80	0.07	0.87	0.02	0.001
4th	s	0.63	0.08	0.60	0.02	0.601	0.48	0.04	0.48	0.01	0.709
5th	s	0.50	0.07	0.46	0.02	0.068	0.66	0.04	0.55	0.02	<0.001
6th	s	0.63	0.07	0.64	0.02	0.541	0.46	0.04	0.46	0.01	0.857
7th	s	0.62	0.07	0.62	0.02	0.800	0.78	0.09	0.80	0.02	0.444

**Figure 2:**
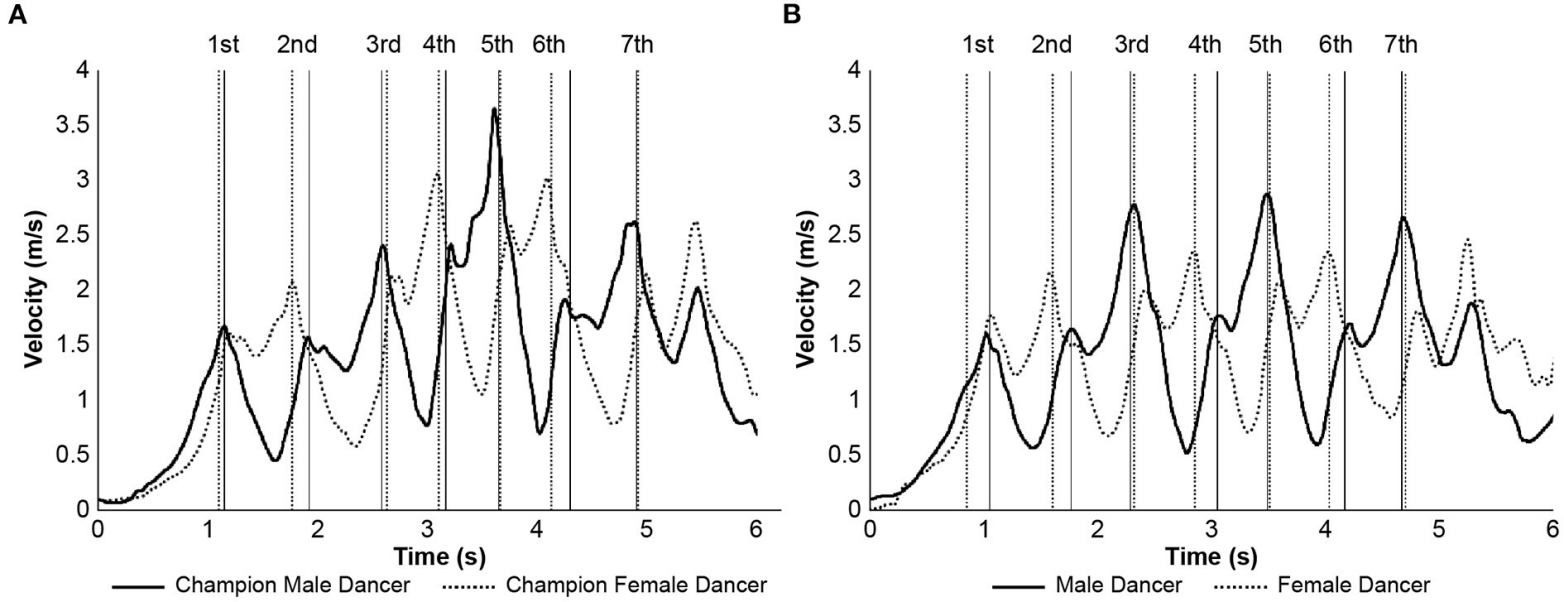
Pelvic segment resultant velocities during the spin movement. The vertical line is the timing when the maximum value of each step for male and female is reached, and solid line is male and dotted line is female. **(A)** The champion male and female dancers. Solid line is male. Dotted line is female. **(B)** The control group male and female dancers. Solid line is male. Dotted line is female.

Figure 2 shows the resultant velocity of the pelvic segments for the champion male and female dancers and representative male and female dancers of the control group. All dancers performed spin movement by repeatedly increasing and decreasing velocities; when the velocity for male dancer increased, female dancer's velocity decreased, while when the male dancer's velocity decreased, the velocity of female dancer increased. Peak velocities were attained at a time when the step length was approximately maximum for both the dancers. Exact peak values were different between the champion dancers and control group dancers. Table 4, based on Figure 2, shows the peak resultant velocity values corresponding to the third and fifth steps for males and the fourth and sixth steps for females. The velocities of the champion male and female dancers were significantly higher than that of the control group male and female dancers, respectively.

**Table 4:** Peak pelvic segment resultant velocities of the champion male and female dancers and the control group male and female dancers during the spin movement.

		**Male dancers**		**Champion male dancer**			**Female dancers**		**Champion female dancer**		
Step	Unit	Mean	SD	Mean	SD	*p*-value	Mean	SD	Mean	SD	*p*-value
3rd for male, 4th for female	m/s	2.18	0.20	2.41	0.03	0.002	2.68	0.23	2.95	0.10	0.001
5th for male, 6th for female	m/s	2.83	0.22	3.65	0.04	<0.001	2.83	0.22	3.05	0.04	0.004

**Figure 3:**
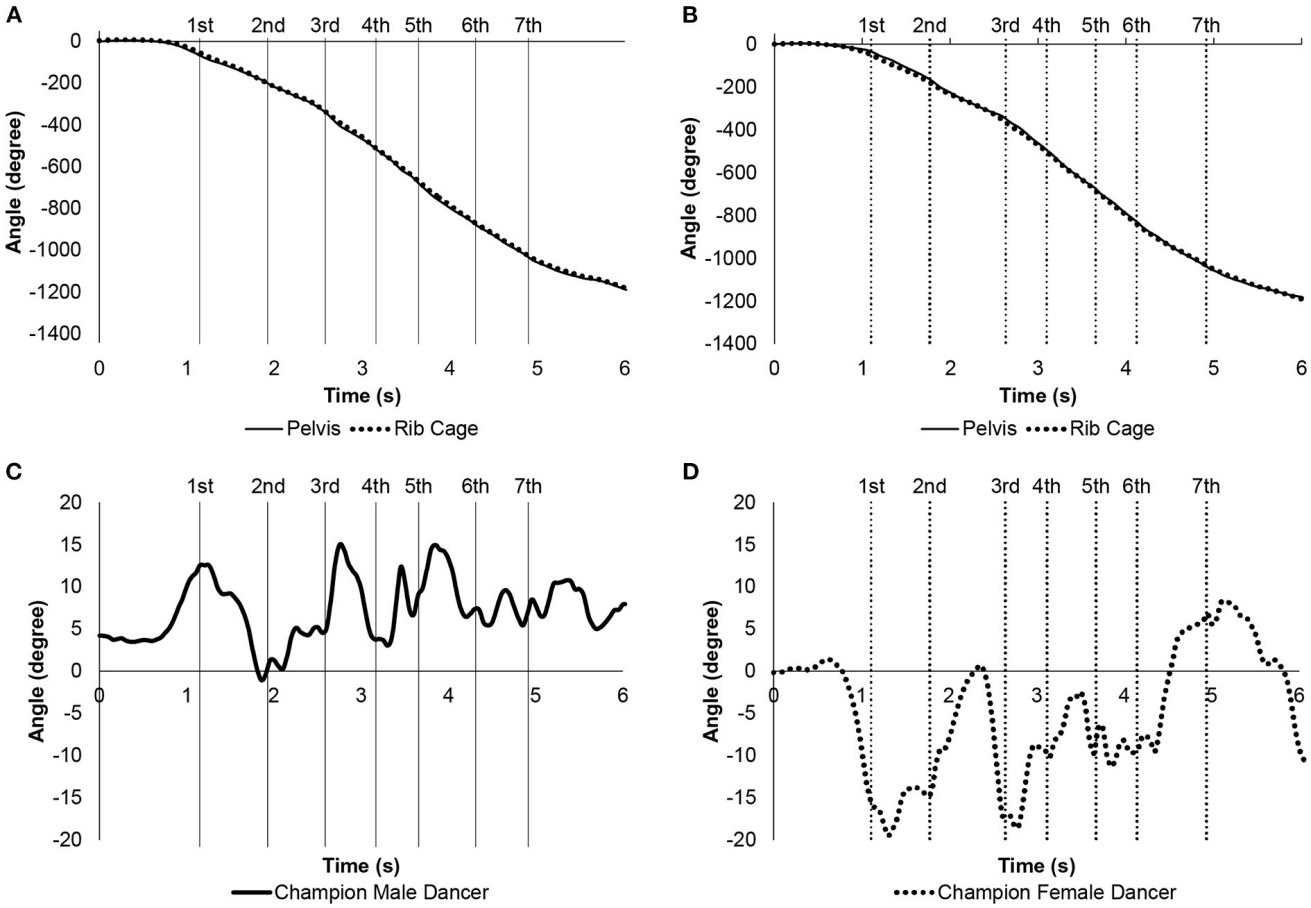
Euler angles of pelvic and rib cage segments in vertical axis for the champion male and female dancers. The vertical line is the timing when the maximum value of each step for the champion male and female dancers is reached, and solid line is male and dotted line is female. **(A)** Angles of the champion male dancer. Solid line is pelvic segment. Dotted line is rib cage segment. **(B)** Angles of the champion female dancer. Solid line is pelvic segment. Dotted line is rib cage segment. **(C)** Relative angles of the rib cage segment with respect to the pelvic segment for the champion male dancer. Positive values at the relative angles indicated that the rib cage segment rotates to the left and negative values rotate to the right with respect to the pelvic segment. **(D)** Relative angles of the rib cage segment with respect to the pelvic segment for the champion female dancer. Positive values at the relative angles indicated that the rib cage segment rotates to the left and negative values rotate to the right with respect to the pelvic segment.

**Figure 4:**
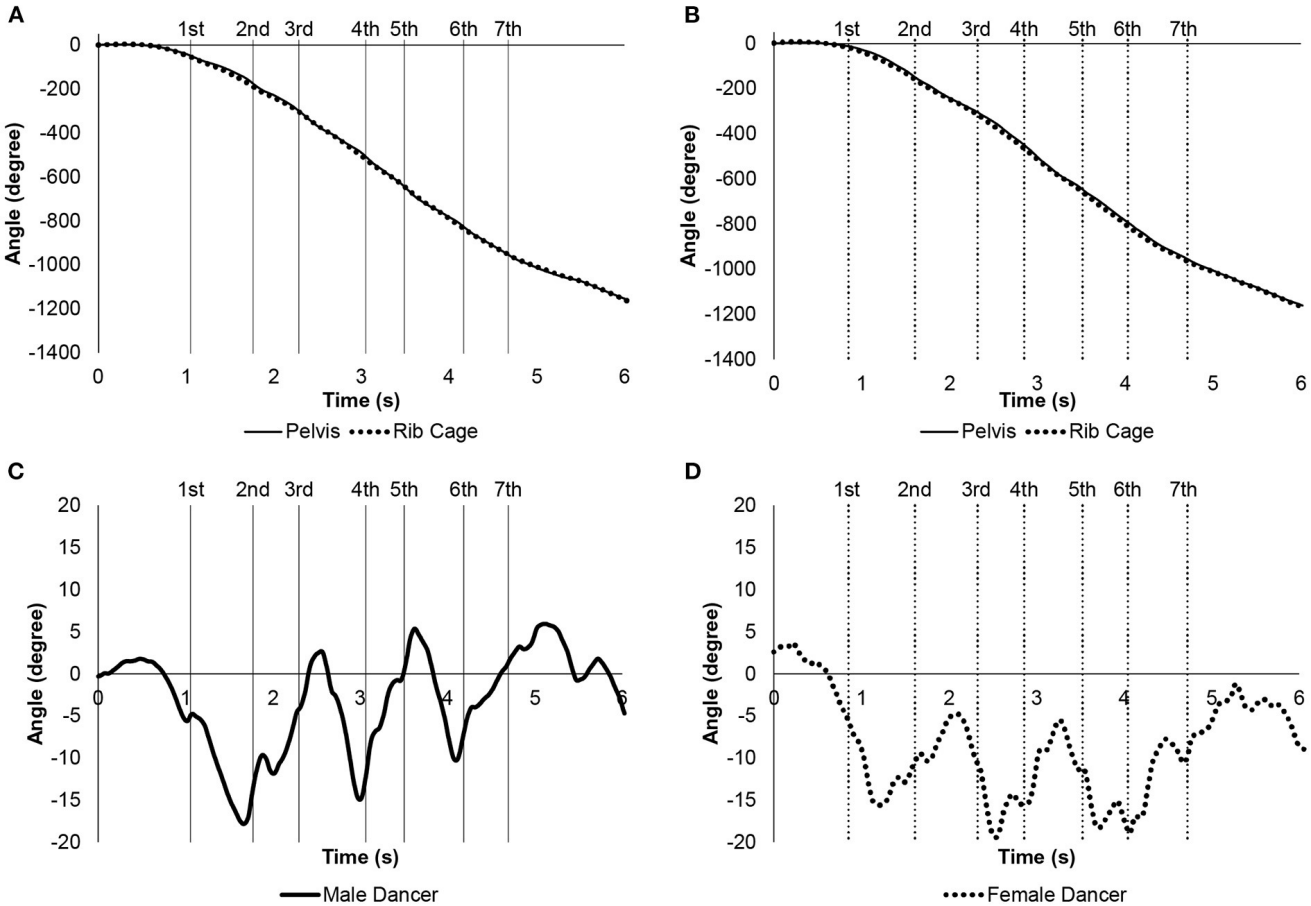
Euler angles of pelvic and rib cage segments in vertical axis, representative example for the control group male and female dancers. The vertical line is the timing when the maximum value of each step for the control group male and female dancers is reached. **(A)** Angles of the control group male dancer. Solid line is pelvic segment. Dotted line is rib cage segment. **(B)** Angles of the control group female dancer. Solid line is pelvic segment. Dotted line is rib cage segment. **(C)** Relative angles of the rib cage segment with respect to the pelvic segment for the control group male dancer. Positive values at the relative angles indicated that the rib cage segment rotates to the left and negative values rotate to the right with respect to the pelvic segment. **(D)** Relative angles of the rib cage segment with respect to the pelvic segment for the control group female dancer. Positive values at the relative angles indicated that the rib cage segment rotates to the left and negative values rotate to the right with respect to the pelvic segment.

Figures 3A,B, 4A,B show the time-series changes in the Euler angles for the vertical axis of the pelvic and rib cage segments in the champion male and female dancers and representative male and female dancers of the control group, respectively. This angle is the rotation of the body on the horizontal plane. The champion male dancer showed rotation of the pelvis before the rib cage segment near the first step, while both the segments rotated at almost the same time near the second step. Then, in the third to seventh steps, the pelvic segment rotated before the rib cage segment as well. In the control group male dancers, the rib cage segment rotated before the pelvic segment near the first and second steps, while the pelvic segment rotated before the rib cage segment near the third step. Further, the fourth to seventh steps involved similar repetitive movements. Among female dancers, the champion showed rotation of rib cage ahead of the pelvic segment in the first step; this was followed by preceding rotations of pelvic and rib cage segments in subsequent steps. The rib cage segment of control group female dancers rotated faster from the first to the last step.

Figures 3C,D, 4C,D show the time-dependent changes in the relative angles for the vertical axis of the rib cage segment with respect to the pelvic segment in the champion male and female dancers and the control group male and female dancers, respectively. Positive and negative values indicated left and right rotations of the rib cage with respect to the pelvic segment, respectively. The champion male dancer showed positive peaks near the first, third, and fifth steps; the champion female dancer showed negative peaks at those instances. Further, the relative angles of the champion male and female dancers tended to change when one increased and other decreased the velocity. Among control group of dancers, male dancers showed positive peaks near the third and fifth steps, with corresponding negative peaks of the female dancers. Interestingly, the relative angles of champion male dancer and control group male dancers were almost positive and negative during the entire assessment duration, respectively.

Table 5 shows the mean values of the relative angle of the rib cage segment in the vertical axis with respect to the pelvic segment from the second to the sixth step in the champion male and female dancers and control group male and female dancers. The angle of the champion male and female dancers was significantly larger than that of the control group male and female dancers (*p* < 0.001 and *p* = 0.027, respectively).

**Table 5:** Mean relative angle of the rib cage segment with respect to the pelvic segment in the vertical axis for the champion male and female dancers and the control group male and female dancers during the spin movement.

		**Male dancers**		**Champion male dancer**			**Female dancers**		**Champion female dancer**		
**Angle**	**Unit**	**Mean**	**SD**	**Mean**	**SD**	***p*** **-value**	**Mean**	**SD**	**Mean**	**SD**	***p*** **-value**
Pelvis –rib cage	Degree	−2.37	4.07	7.10	0.81	<0.001	−10.72	5.78	−6.68	1.27	0.027

*Positive and negative values at the relative angles indicated that the rib cage segment rotated to the left and right, respectively, with respect to the pelvic segment*.

## Discussion

This study investigated the kinematic characteristics of the world champion male and female dancers during spin movement in competitive waltz, also known as English waltz or slow waltz.

We identified characteristic variables of Euler angles on the vertical axis of the pelvic and rib cage segments associated with the closed-hold position. In addition, we identified the pelvic resultant velocity, step length, duration and heel height at the maximum step length, which are related to speedy movements. Significant differences in these variables were observed between the champion male and female dancers and the male and female dancers in the control group. These results could be used to evaluate performance and serve as a reference for daily practice for competitive ballroom dancers and coaches.

The characteristics of movements for the closed-hold position can be understood from the tendency of the change in step length from the start step to the seventh step. The change appears in both the absolute step length and the normalized step length. Basically, there is no linear movement like walking in competitive ballroom dancing. Therefore, the distance between the left and right ankle joints was defined as the step length. In male dancers for the control group and the champion male dancer, the first, third, fifth, and seventh steps moved more than the second, fourth, and sixth steps. On the other hand, the step length of the female dancers in the control group and the champion female dancer showed the opposite pattern. In the closed-hold position, while males maintain the head and the upper body in an upright position during the hold, females perform a lateral flexion and a hyperextension both in the trunk and neck to increase the aesthetic appearance (Vaczi et al., 2016). In addition, the right halves of the male and female overlap because of the closed-hold position. Therefore, for the dancer's right leg to move forward, it is necessary to place it between the partner's legs. In other words, in the closed-hold position, the right leg is difficult to move, and conversely, the left leg moves easily without any obstacles. However, the step length was longer during the spin movement when the left leg became the leading leg.

Significant differences in both actual and normalized step length results indicate the characteristics of the movement of the world champion dancers. Because height and leg length were not taken into consideration in the competition, it became clear that the actual step lengths of the champion male and female dancers were remarkably long. The world's top-ranked competitive ballroom dancers have participated in experiments in previous research on exercise physiology (Liiv et al., 2014). Compared to the heights of these participants, they were similar in height to the world champion male and female dancers. However, the heights of the male and female dancers in the control group in this study were considerably lower than those of the participants. Competitive ballroom dancing seems to require the ability to move with large step lengths. Therefore, the length of the lower limb and the ability to control the lower limb during the closed-hold position to produce a larger step length can be considered as one of the important factors for assessing performance.

Features appeared in some variables in the results of the height of the left and right heels at each step timing. In this study, calibration is performed while wearing shoes according to the manual of Xsens system. The system only inputs the length of the foot and the height to the ankle, and does not take into account the high heel shoes used by female dancers. Therefore, the shoe and the foot are integrated to form the foot segment. In this respect, unlike the optical method, the IMU method does not have a reflection marker attached to the heel, so care must be taken in understanding the data. Competitive ballroom dancing basically does not raise heels without moving like ballet. A value as close to zero as possible means that the heel is on the ground for the male dancers and the tip of the heel is on the ground for the female dancers. It can be interpreted that the value around 0.1 m supports the body near the toes and forefoot. In the slow movement of the first half, the heel of the champion male dancer tended to be higher than that of the control male dancers. However, in the fast movements in the latter half, the difference between the champion male dancer and the control male dancers tended to be unclear. In the slow movement, the characteristic of the champion male dancer who has a high ability to raise the heel came out. On the other hand, it is probable that it was difficult to make a difference because the time to keep raising the heel was short with fast movement. The characteristics of the champion female dancer are far from clear. Since competitions in which dancers wear high heels are rare, further research, including measurement methods, is needed.

The control group male and female dancers had a longer duration in the first step, while the champion male and female dancers showed a longer duration in the second and third steps. The reason why the male and female dancers had a longer first step because of multiple ways to start, and this variety was reflected in the standard deviation of the first step of male and female dancers of the control group. Here, the champion male and female dancers moved slowly at the beginning of the spin movement. A dance is not just a physical exercise, but a physical expression that matches music and is related to art. It is difficult to understand the artistry of dance movements for biomechanical analysis, but it can be inferred that the champion male and female dancers adopted a strategy based on artistry.

The increase and decrease in the resultant velocity of the pelvic segment represents the characteristics of spin movement. In a couple performing dance, when the velocity of one of the partners increased, the velocity of other partner decreased. At the start, the female was in the forward direction, and the male was in the backward direction with a closed-hold position. Subsequently, from this posture, it moved forward while rotating clockwise. Therefore, it is necessary for the forward dancer to be standing left to the direction of movement in order to overtake the backward dancer standing to the right. In other words, the velocity difference between dancers is essential for overtaking. Previous studies have reported that top-ranked dancers were faster than that of the lower-ranked dancers in clockwise rotation (Prosen et al., 2013). Although dance music was different in this study, the same results were obtained. In previous research, the trajectory of a dancer was measured using a computer vision tracking algorithm (Zaletel et al., 2010), but the details of the movements could not be measured. In this study, we succeeded in obtaining detailed information on body movements using a IMUs system.

The peak resultant velocity of the pelvic segment was distinctively higher in the champion male and female dancers than that of the control group male and female dancers. Among them, the velocity of the fifth step of the champion male dancer was remarkable. In addition to the time-series change, extracting the peak values from it is an index showing speedy movement. From the velocity of the time-series change from the first step to the seventh step, the champion male and female dancers gradually increased the velocity from the start. It seems that the champion male and female dancers strategically reached the maximum velocity in the fifth step.

During spin movement, the dancer rotates clockwise with a closed-hold position. Therefore, it may be important to consider the Euler angle of the pelvic and rib cage segments along the vertical axis and the relative angle of the rib cage segment with respect to the pelvic segment, consisting of the trunk in the vertical axis direction. Positive peaks appeared for the male dancers and the champion male dancer and negative peaks for the female dancers and the champion female dancer near the third and fifth steps in the relative angles. The male dancers and champion male dancer had peaks in the velocity of the pelvic segment at the third and fifth steps. In accordance with this timing, the rib cage segment rotated greatly to the right for the female dancers and the champion female dancer and greatly to the left for the male dancers and the champion male dancer with respect to the pelvic segment. This characteristic phenomenon may occur when the ballroom dancers rotate quickly clockwise, such as in the spin movements, while maintaining a closed-hold position. However, there was a peak in the relative angles of the male and female champion dancers, even in the first step. The peak of the first step can be considered to indicate that the champion male and female dancers can maintain the positional relationship between the pelvic and rib cage segments in the closed-hold position, even as they begin to move slowly.

Of particular interest is that the mean values of the relative angles of the rib cage segment with respect to the pelvic segment for the champion male dancer were positive, whereas those of the others were negative. By calculating the average value as well as the time-series change, the posture that serves as the reference for the closed-hold position becomes clear. When considered as a couple, the champion male and female dancers had the rib cage segment rotated more to the left with respect to the pelvic segment than that of the male and female dancers. The spin movement rotates clockwise, that is, a motion that rotates to the right. Thus, the strategy of rotating the rib cage segment to the right seems reasonable, but the champion male dancer did not. The mechanical mechanism was not understood in this study. However, it can be predicted that it is probably necessary to move at a higher speed without breaking the closed-hold position. There are technical terms in ballroom dancing that describe the turning movement, such as “contrary body movement,” “rotation” and so on. These terms probably suggest that turning movements are important for successful ballroom dancing. The details of the holding postures of the world champion couple in this study during turns will be useful information for competitive dancers and coaches. Moreover, because the pelvic and rib cage segments are twisted, muscle stretching may also be involved (Komi, 2000; Fukutani and Herzog, 2021). Incorporating not only the mechanical part of competitive ballroom dancing but also the viewpoint of muscle physiology will lead to an evaluation of the performance of competitive ballroom dancers.

In addition to the difference in the positional relationship between the pelvic and rib cage segments in the closed-hold position, the timing of rotation of the pelvic and rib cage segments of the champion male dancer was considerably different from that of other dancers. The champion male dancer first moved the pelvic segment followed by the rib cage segment. The other dancers moved in the reverse order. The movement of the pelvic and rib cage segments suggests that the male and female dancers and the champion female dancer lead the rib cage segments to perform spin movements. In contrast, the male champion dancer moved the pelvic segment ahead of the rib cage segment. This particular move may be related to the effective use of the lower limb muscles.

This study had some limitations and advantages. First, the kinetic analyses were not performed. Second, the ground reaction and contact forces between the dancers could not be measured. The combination of kinematic and kinetic information makes the movements during the competitive ballroom dancing more understandable. However, the advantage of this study was that kinematic information was obtained on competitive ballroom dancing. Optical motion capture systems (Topley and Richards, 2020) are widely used to understand details for many kinds of movements. The optical system does not allow uniform visualization because the male and female dancers are in a closed-hold position. Therefore, in this study, IMUs system (Pedro et al., 2021) was used to measure variables peculiar to competitive ballroom dancing.

This study also has some limitations and advantages for the experimental participants. One of the strengths was that we were able to measure the world champion dance couple at the time of the measurement. There was only one world champion dance couple, and kinematic differences between previous and current champion dance couples were not clear. Although top-ranked dance couples at the national-level participated as a control group, kinematic differences with dance couples at the international-level were not evident. In addition, it is considered that there are global regional differences at the national-level as well. This study compares the world champion couple and the control group with different anthropometric characteristics and dance experiences. It is not clear how these differences relate to kinematic characteristics. Despite these limitations, little biomechanics research has been conducted on competitive ballroom dancing, and the results obtained from this research are valuable. The results obtained will be useful for daily practice for competitive ballroom dancers and coaches.

In conclusion, this study revealed the kinematic characteristics of the champion and top-ranked male and female dancers when they performed spin movement in the competitive ballroom dancing. There are technical and artistic aspects to competitive ballroom dancing. Biomechanics research has revealed the following, although it is limited to the technical aspects. In comparison to the national-level top dancers, the champion dancers showed a larger step length and faster pelvic velocity. In addition, large differences were observed in the movements of the pelvic and rib cage segments and the relative angle of the rib cage segment to the pelvic segment during the closed-hold position. The champion male dancer started to move from the pelvic segment, whereas the champion female dancer and the national-level top male and female dancers started to move from the rib cage segment. During the spin movement, the champion male dancer was in a position where the rib cage segment was rotated to the left with respect to the pelvic segment, whereas the other dancers were in a position where the rib cage segment was rotated to the right. These findings on the characteristics of the holding posture and lower limb movements would be considered part of the variables to evaluate performance in competitive ballroom dancing.

## Data availability statement

The datasets analyzed in this study are not publicly available. Requests to access the datasets should be directed to Yasuyuki Yoshida, yasuyuki.yoshida@aist.go.jp.

## Ethics statement

The studies involving human participants were reviewed and approved by the Committee for Ergonomic Experiments, National Institute of Advanced Industrial Science and Technology (No. 2018-0823), Tokyo, Japan.

## Author contributions

YY and TN contributed to the conception and design of this study. SN and RN contributed to the data collection. AB, KD, SN, and RN contributed to the data collection and provided advice on the interpretation of the results. All authors contributed to the article and approved the submitted version.

## Funding

This paper is based on the results obtained from a project, JPNP20006, commissioned by the New Energy and Industrial Technology Development Organization (NEDO). This study was also supported by KAKENHI (22K11491).

## Conflict of interest

The authors declare that the research was conducted in the absence of any commercial or financial relationships that could be construed as a potential conflict of interest.

## Publisher's note

All claims expressed in this article are solely those of the authors and do not necessarily represent those of their affiliated organizations, or those of the publisher, the editors and the reviewers. Any product that may be evaluated in this article, or claim that may be made by its manufacturer, is not guaranteed or endorsed by the publisher.
